# Characteristics and Symptoms of App Users Seeking COVID-19–Related Digital Health Information and Remote Services: Retrospective Cohort Study

**DOI:** 10.2196/23197

**Published:** 2020-10-20

**Authors:** Amichai Perlman, Alina Vodonos Zilberg, Peter Bak, Michael Dreyfuss, Maya Leventer-Roberts, Yael Vurembrand, Howard E Jeffries, Eyal Fisher, Yael Steuerman, Yinat Namir, Yaara Goldschmidt, Daniel Souroujon

**Affiliations:** 1 K Health Inc New York, NY United States; 2 Icahn School of Medicine at Mount Sinai New York, NY United States; 3 Center for Corona Treatment Maccabi Health Services Tel Aviv Israel; 4 Department of Pediatrics Seattle Children's Hospital University of Washington School of Medicine Seattle, WA United States; 5 Li Ka Shing Centre Cancer Research UK Cambridge Institute University of Cambridge Cambridge United Kingdom

**Keywords:** digital health, remote care, symptom checker, telemedicine, COVID-19, symptom, cohort study, self-reported, online tool

## Abstract

**Background:**

Patient-facing digital health tools have been promoted to help patients manage concerns related to COVID-19 and to enable remote care and self-care during the COVID-19 pandemic. It has also been suggested that these tools can help further our understanding of the clinical characteristics of this new disease. However, there is limited information on the characteristics and use patterns of these tools in practice.

**Objective:**

The aims of this study are to describe the characteristics of people who use digital health tools to address COVID-19–related concerns; explore their self-reported symptoms and characterize the association of these symptoms with COVID-19; and characterize the recommendations provided by digital health tools.

**Methods:**

This study used data from three digital health tools on the K Health app: a protocol-based COVID-19 self-assessment, an artificial intelligence (AI)–driven symptom checker, and communication with remote physicians. Deidentified data were extracted on the demographic and clinical characteristics of adults seeking COVID-19–related health information between April 8 and June 20, 2020. Analyses included exploring features associated with COVID-19 positivity and features associated with the choice to communicate with a remote physician.

**Results:**

During the period assessed, 71,619 individuals completed the COVID-19 self-assessment, 41,425 also used the AI-driven symptom checker, and 2523 consulted with remote physicians. Individuals who used the COVID-19 self-assessment were predominantly female (51,845/71,619, 72.4%), with a mean age of 34.5 years (SD 13.9). Testing for COVID-19 was reported by 2901 users, of whom 433 (14.9%) reported testing positive. Users who tested positive for COVID-19 were more likely to have reported loss of smell or taste (relative rate [RR] 6.66, 95% CI 5.53-7.94) and other established COVID-19 symptoms as well as ocular symptoms. Users communicating with a remote physician were more likely to have been recommended by the self-assessment to undergo immediate medical evaluation due to the presence of severe symptoms (RR 1.19, 95% CI 1.02-1.32). Most consultations with remote physicians (1940/2523, 76.9%) were resolved without need for referral to an in-person visit or to the emergency department.

**Conclusions:**

Our results suggest that digital health tools can help support remote care and self-management of COVID-19 and that self-reported symptoms from digital interactions can extend our understanding of the symptoms associated with COVID-19.

## Introduction

Contemporary health care systems have limited capacity for managing epidemics; they are structured on the model of in-person interactions between patients and clinicians, which results in the congregation of patients in emergency departments and waiting areas during crises [1]. During the COVID-19 pandemic, it has been suggested that digital technologies can be used to deliver rapid urgent assessments, aid management of nonurgent conditions, and reduce the risk of iatrogenic COVID-19 exposure [1-3]. Indeed, several medical associations have encouraged the use of these tools during the COVID-19 epidemic, including the American Medical Association and the American Academy of Family Physicians [4,5]. Moreover, it has been suggested that these technologies can provide novel insights regarding the epidemiology and clinical characteristics of this new disease [6], especially in early stages and in community settings, as most published studies focus on hospitalized patients [7].

Digital tools have been promoted and deployed during previous infectious disease outbreaks, such as severe acute respiratory syndrome (SARS) and Ebola virus [8,9]. These tools focused primarily on surveillance, contact tracing, case management, and management of laboratory results [10]. In recent years, a wide range of patient-facing digital health modalities have emerged. These include protocol-based triage tools, artificial intelligence (AI)–driven tools for diagnosis and self-assessment, use of at-home remote monitoring devices, and virtual consultations by remote physicians. These tools are rapidly being deployed to support the medical management of the current epidemic [2]. However, despite the rapid growth in patient-facing digital and AI-driven technologies, other than reports of user satisfaction [11-13], there are limited data on the usage patterns and characteristics of these technologies in the management of COVID-19 and on their utility for advancing clinical research of COVID-19. Examining the characteristics and symptoms of digital technology users can help identify novel features associated with positivity or severity of COVID-19 and advance our understanding of the disease. Analysis of use patterns of digital tools can provide insight regarding their utility in the management of COVID-19 and can also highlight opportunities for further development.

During the pandemic, K Health, a novel AI-driven digital health platform deployed in the United States [14], offered three tools: a protocol-based COVID-19 self-assessment tool, an AI-driven symptom checker, and text–based telemedicine visits. This study aims to further our understanding of the use of digital health technologies in COVID-19 management and research. Specifically, the study describes the demographic and clinical characteristics of people using digital health tools for COVID-19–related concerns, explores self-reported symptom patterns among digital health users with COVID-19 compared to users who do not have the disease, and characterizes the information and management recommendations provided by different digital health tools.

## Methods

### Population and Settings

K Health is a novel AI-driven digital health app; at the time this paper was written, it was available for download in the United States, Mexico, Indonesia, and Israel [14]. The app was built using a data-driven approach in collaboration with Maccabi Health Services (MHS). MHS is the second largest health maintenance organization in Israel, with over 2 million members [15,16]. Data from the MHS health records were used to develop an AI-driven symptom-checker, whose methodology has been described elsewhere [14]. The K Health app was launched in the United States in 2018 and has been used by over 4 million adults [14]. The app is free to download, and the symptom checker is available for free to adults over 18 years of age; thus, the app provides the public with reliable and personalized information on diagnoses related to their symptoms and medical history.

In June 2019, K Health began offering a service that enables users to consult directly with a remote physician. The platform provides the users with the option to receive a diagnosis, prescriptions, lab referrals, a referral to remote management of mental health concerns, or a referral to primary or emergency care. Consulting with a remote physician involves a fee or enrollment in a relevant health plan.

Early in April 2020, a protocol-based COVID-19 self-assessment was added to the app to provide users with up-to-date guidance for managing suspected cases of COVID-19. The self-assessment was developed by a team of board-certified physicians and was based on guidance issued by the World Health Organization and the US Centers for Disease Control and Prevention [17,18]. The user was asked to report the presence and severity of COVID-19–related symptoms as well as the presence of concomitant conditions known to increase risk of severe disease. Following the self-assessment, users were provided with one of four recommended actions based on their risk profile: social distancing, quarantine, isolation, or seeking immediate medical evaluation. Users were also informed if they were at increased risk for COVID-19 complications, and users with risk factors and symptoms were encouraged to consult a physician. For further details, including the full questionnaire, see Multimedia Appendix 1.

### Study Design

This study describes use of three digital tools, namely a protocol-based COVID-19 self-assessment tool, an AI-driven symptom checker, and communication with a remote physician, by adults (>18 years of age) seeking COVID-19–related health information and services during the 10-week period between the launch date of April 8 to the date of data extraction on June 20, 2020. Seeking COVID-19–related health information was defined as use of the COVID-19 self-assessment tool. This self-assessment tool can be activated by the user, and it is also activated automatically when users begin a symptom check with a complaint of cough, dyspnea, or fever. The study did not include repeated self-assessments.

After users perform the COVID-19 self-assessment, they are prompted to use the AI-driven symptom checker to receive additional information about other conditions that may be related to their symptoms. After receiving the results and information regarding these conditions, the users can choose to communicate with a remote physician for definitive medical management.

This study analyzed deidentified data. The protocol of the study was reviewed by the Western Institutional Review Board and qualified for exemption in accordance with 45 CFR § 46.104(d)(4). K Health is compliant with the Health Insurance Portability and Accountability Act (HIPAA) and the General Data Protection Regulation (GDPR). Encrypted transportation and storage were used at all stages of data management.

### Variables

Deidentified data on the characteristics of the digital interactions in the app were collected, including user-inputted demographic and clinical characteristics; COVID-19 exposure reporting; reports of COVID-19 testing, including results; and symptoms. We also collected the outputs provided by the three digital modalities: risk categories according to the protocol-based COVID-19 self-assessment; the most common conditions for similar people and symptoms (“potential diagnoses”) provided by the AI-driven symptom checker; and diagnoses, management, and disposition by remote physicians.

User characteristics included age, sex, risk factors, and comorbidities. Comorbidities included hypertension, smoking, obesity, diabetes (type 1 and type 2), cardiovascular disease, chronic lung disease (asthma, chronic obstructive pulmonary disorder, and interstitial lung disease), chronic renal and liver disease, cancer, and immune suppression.

Symptoms were obtained from two types of interactions on the platform: first, as part of the structured protocol-based COVID-19 risk assessment, and second, as part of the dynamic AI-driven health dialog.

The COVID-19 self-assessment included questions regarding symptoms known to be associated with COVID-19 (eg, cough, dyspnea, and fever) as well as symptoms hypothesized to be related to COVID-19 (eg, eye symptoms), which were inquired about for further research and development of the COVID-19 protocol. The symptom checker dialog allows the user to spontaneously discuss a wider array of 331 symptoms; it also queries the user regarding additional symptoms and symptom-specific attributes, such as the severity, timing, duration, and quality of a symptom (eg, dry or productive cough), as previously described [6].

### Analyses

The analyses assessed the user characteristics, reported symptoms, and output of the aforementioned digital services, namely the self-assessment protocol, AI- and data-driven symptom checker, and remote physician consultation. This assessment was performed using descriptive statistics and bivariate analyses.

Descriptive univariate summary statistics were developed to assess the user characteristics, reported symptoms, diagnoses, and disposition. Symptoms were assessed for both the COVID-19 self-assessment and the symptom checker. Bivariate analyses were conducted for between-group comparisons of characteristics and symptoms. These included two primary analyses.

The first analysis explored the potential utility of self-reported data from digital health tools to identify symptoms associated with COVID-19. This analysis compared the self-reported symptoms by digital health users who reported testing positive for COVID-19 with those of users who reported testing negative. To ensure that the symptoms of cough, fever, and dyspnea were optimally captured, the presence of these symptoms was collected from both the self-assessment and the symptom checker dialog. More details are provided in Multimedia Appendix 1.

The second analysis aimed to identify predictors related to the choice to consult with a remote physician. This analysis compared data provided by individuals who opted to consult with a remote physician with those provided by the entire cohort. Specifically, this comparison aimed to evaluate whether consultation with a physician was related to risk of COVID-19, symptom severity, or comorbidity.

Appropriate statistical tests were used to assess the significance of between-group differences: chi-square tests for differences in proportions and *t* tests for differences in continuous measures. Test assumptions were assessed analytically and graphically and were judged to be adequately met. We considered *P* values of .05 or less to be significant and did not correct for multiple comparisons. Analyses were conducted using Python version 3.6.9.

## Results

**Figure 1:**
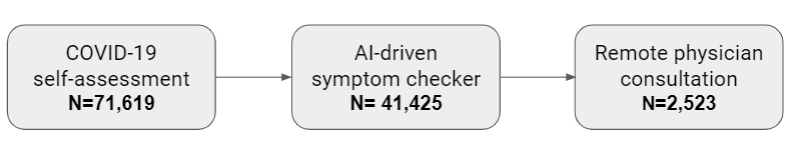
Flowchart describing the cohort of US individuals who used the COVID-19 self-assessment between April 8 and June 20, 2020. AI: artificial intelligence.

During the assessed timeframe, 71,619 individuals completed the COVID-19 self-assessment. The self-assessment included questions on COVID-19 exposure, testing, comorbid conditions, and the presence of COVID-19–related symptoms. The self-assessment output provided users with protocol-based information and recommendations on their COVID-19 risk. Of the 71,619 individuals who completed the self-assessment, 41,425 (57.8%) also completed a more detailed AI-driven symptom checker. The symptom checker provides the option to evaluate a wide range of 331 symptoms and to receive information about non–COVID-19 conditions that may be related to these symptoms. Following use of the symptom checker, a subset of 2523 users proceeded to consult with remote physicians, who provided guidance on the users’ disposition and health management (Figure 1). 

Individuals using the COVID-19 self-assessment were predominantly female (51,845/71,619, 72.4%), with a mean age of 34.5 years (SD 13.9). The most commonly reported comorbidities were chronic lung disease, primarily asthma (16,080/71,619, 22.5%), and hypertension (13,952/71,619, 19.5%) (Table 1).

The self-assessment results provided users with information and recommendations related to their risk of COVID-19, with the most common recommendations involving social distancing (20,984/71,619, 29.3%) and isolation (23,706/71,619, 33.1%) (Table 2).

**Table 1:** Characteristics of the cohort of COVID-19 self-assessment users (N=71,619).

Characteristic		Value
**Demographics**		
	Age, mean (SD)	34.5 (13.9)
	Female, n (%)	51,845 (72.4)
**Chronic conditions, n (%)**		
	Hypertension	13,952 (19.5)
	Morbid obesity	7676 (10.7)
	Smoking	13,505 (18.9)
	Stroke	897 (1.3)
	Cancer or immunosuppression	1868 (2.6)
	Chronic kidney disease	1313 (1.8)
	Chronic lung disease	16,080 (22.5)
	Cardiovascular disease	3835 (5.4)
	Diabetes	5958 (8.3)
**Tested for COVID-19 (n=2901)** **, n (%)**		
	Tested positive	433 (14.9)
	Tested negative	2468 (85.0)

**Table 2:** Outputs provided by the digital health tools (N=71,619), n (%).

Output			Value
**COVID-19 assessment recommendation**			
	Practice social distancing	20,984 (29.3)	
	Isolate yourself	23,706 (33.1)	
	Quarantine yourself	7735 (10.8)	
	Seek immediate evaluation	19,194 (26.8)	
**Most common symptom checker potential diagnoses (n=41,425)**			
	Upper respiratory infection	15,232 (36.7)	
	Anxiety disorder	2457 (5.9)	
	Gastroesophageal reflux disease	2075 (5.0)	
	Dehydration	1512 (3.6)	
	Tension-type headache	1152 (2.8)	
	Allergic rhinitis	957 (2.3)	
	Depressive mood disorder	833 (2.0)	
	Pulmonary embolism	783 (1.9)	
	Pneumonia	721 (1.7)	
	Acute food poisoning	708 (1.7)	
**Remote physician management and disposition^a^** **(n=2523)**			
	Medication or laboratory tests	1072 (42.5)	
	Information and reassurance	431 (17.1)	
	Other	363 (14.4)	
	Referral to primary care or a specialist	325 (12.9)	
	Referral to the emergency department	257 (10.2)	
	Referral to COVID-19 testing	169 (6.7)	
	Referral to a remote behavioral health service	73 (2.9)	

^a^Dispositions are not mutually exclusive.

**Figure 2:**
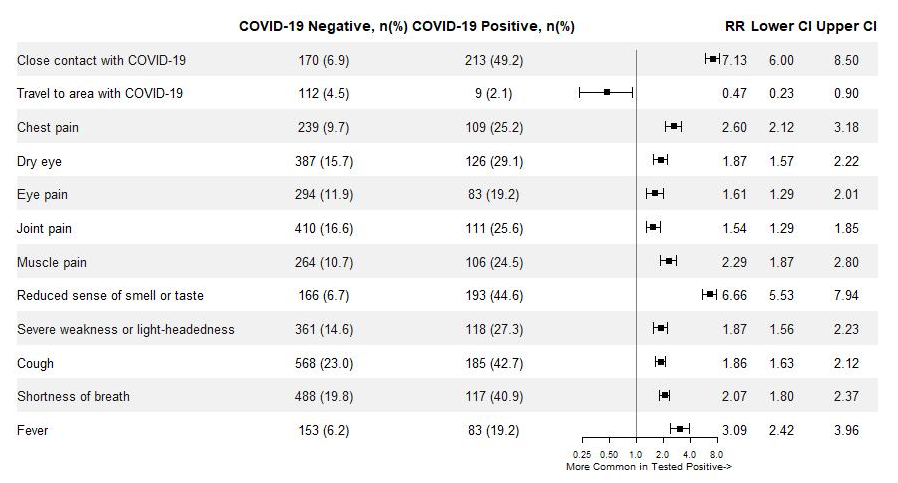
Forest plot presenting the RRs and 95% CIs of each item in the self-assessment questionnaire among users who reported testing positive for COVID-19 compared to those who reported testing negative. Muscle pain was not present in early versions of the self-assessment. Proportions were calculated for self-assessments where the symptom was reportable. RR: relative rate.

Testing for COVID-19 was reported by 2901 users, of whom 433 (14.9%) reported testing positive. Users who were tested for COVID-19 were predominantly female (2022/2901, 69.7%), with a mean age of 38.1 years (SD 13.8). Of users who were tested, those who reported testing positive were of similar age (mean 37.5 years, SD 13.8, vs mean 38.2 years, SD 13.8, *P*=.32), less frequently female (62.4% vs 69.9%), and much more likely to have reported a close contact with a known case of COVID-19 (relative rate [RR] 7.13, 95% CI 6.00-8.49), compared to those who tested negative. The symptoms reported by users as part of the COVID-19 self-assessment differed between those who reported positive and negative test results. Most notably, the relative prevalence of reporting loss of smell or taste was significantly higher among individuals who tested positive for COVID-19 (RR 6.65, 95% CI 5.53-7.94), as was the prevalence of fever (RR 2.58, 95% CI 2.04-3.26) and chest pain (RR 2.59, 95% CI 2.12-3.18). Cough, difficulty eating or drinking, dry eye, eye pain, feeling weak and lightheaded, and chest pain were also significantly associated with positive COVID-19 tests (Figure 2).

Individuals using the COVID-19 self-assessment were prompted to use the AI-driven symptom checker to receive additional information about other conditions potentially related to their symptoms. This tool enables users to input a wide range of symptoms that are not systematically collected as part of the COVID-19 self-assessment; thus, it recorded a wide range of additional symptoms that were not already included in the protocol-based self-assessment. Among these, the most common additional symptoms reported by individuals who tested positive for COVID-19 were headache, sore throat, fatigue, diarrhea, chest pain, nausea, nasal congestion, runny nose, and sweating (Supplementary Table 1, Multimedia Appendix 1). The AI-driven algorithm provided users with information about the most common conditions people with symptoms and personal characteristics similar to theirs were diagnosed with (“potential diagnoses”). The most common non–COVID-19 conditions classified as relevant for the 41,425 users were upper respiratory infection (15,232, 36.7%), anxiety disorder (2457, 5.9%), gastroesophageal reflux disease (GERD) (2075, 5.0%), and dehydration (1512, 3.6%) (Table 2).

Among individuals initially evaluating their COVID-19 risk, a small subset (2523/41,425, 3.5%) chose to communicate with a remote physician. Users who chose to communicate with a remote physician were older (mean age 38.6 years, SD 13.2, vs mean age 34.3 years, *P*<.001) and more likely to be male (37.9% vs 27.2%, *P*<.001) than users who did not; however, they were less likely to have comorbidities (RR 0.88, 95% CI 0.82-0.95). These individuals were more likely to report severe symptoms such as severe chest pain (RR 1.13, 95% CI 1.03-1.25) and to be encouraged by the self-assessment to seek immediate evaluation (RR 1.19, 95% CI 1.02-1.32). Additionally, features and symptoms supporting higher suspicion of COVID-19 were more common among users who communicated with a remote physician, including exposure to a close contact with COVID-19 (RR 1.21, 95% CI 1.1-1.35), fever (RR 1.56, 95% CI 1.43-1.72), shortness of breath (RR 1.25, 95% CI 1.16-1.33), and loss of smell and taste (RR 1.22, 95% CI (1.09-1.37) (Table 3).

**Table 3:** User characteristics and self-assessment protocol disposition associated with consulting a remote physician (N=71,619).

Item or disposition			Did not consult a remote physician (n=69,096), n (%)		Consulted a remote physician (n=2523), n (%)		RR^a^ (95% CI)
**Self-assessment items**							
	Close contact	7431 (10.8)		331 (13.1)		1.21 (1.10-1.35)	
	Travel	1908 (2.8)		59 (2.3)		0.82 (0.66-1.09)	
	Eye pain	6257 (9.1)		218 (8.6)		0.95 (0.84-1.09)	
	Dry eye	7242 (10.5)		269 (10.6)		1.02 (0.93-1.17)	
	Joint pain	61,741 (89.4)		2271 (90.0)		1.01 (0.83-1.06)	
	Loss of smell or taste	6051 (8.8)		270 (10.7)		1.22 (1.09-1.37)	
	Feeling weak/light-headed	11,101 (16.1)		462 (18.3)		1.14 (1.05-1.24)	
	Chest pain	8279 (12.0)		343 (13.6)		1.13 (1.03-1.26)	
	Muscle pain	7643 (11.1)		289 (11.5)		1.04 (0.93-1.16)	
	Cough^b^	21,832 (31.60)		859 (34.05)		1.07 (1.02-1.14)	
	Shortness of breath^b^	14,424 (20.8)		656 (26.0)		1.25 (1.16-1.33)	
	Fever^b^	7147 (10.34)		409 (16.21)		1.57 (1.43-1.72)	
	Testing for COVID-19	660 (1.0)		34 (1.3)		1.30 (1.00-2.00)	
**Disposition of self-assessment**							
	Practice social distancing	17,827 (25.8)		777 (30.8)		1.19 (1.15-1.13)	
	Isolate yourself	24,118 (34.9)		781 (30.9)		0.89 (0.67-0.96)	
	Quarantine yourself	9016 (13.1)		176 (6.9)		0.53 (0.34-0.65)	
	Seek immediate evaluation	18,135 (26.3)		789 (31.3)		1.19 (1.02-1.32)	

^a^RR: relative rate.

^b^Users could report these symptoms at the end of the self-assessment or as part of the symptom-checker dialog. See Multimedia Appendix 1 for details.

The remote physicians provided a wide range of evaluation and counseling services, including assessing severe cases and referring them to the emergency department (257/2523, 10.2%) or to ambulatory care in the community (325/2523, 12.9%), advising on and prescribing medications (1072/2523, 42.5%), providing remote behavioral health services to individuals with mild to moderate anxiety or depression (73/2523, 2.9%), providing additional information and reassurance (431/2523, 17.1%), and referring users to COVID-19 testing (169/2523, 6.7%) (Table 2).

## Discussion

### Principal Findings and Interpretation

This study describes the characteristics and management of >71,000 individuals who used digital tools to seek health information and services related to COVID-19. These individuals were relatively young and predominantly female. Users received information regarding COVID-19 risk and management, received AI-driven information about other relevant diagnoses, and consulted with remote physicians. Users who chose to communicate with a remote physician were more likely to have been classified as requiring immediate medical evaluation by the COVID-19 self-assessment. Correspondingly, these users were also older, more likely to report severe symptoms, and more likely to report characteristics cognate with risk of COVID-19 (such as known exposure to COVID-19 and loss of sense of smell or taste). The majority of consultations with a remote physician (1940/2523, 76.9%) were resolved without need for referral to an in-person health visit or to the emergency department.

Taken together, the differential communication with remote physicians according to symptom severity and the high resolution rates without need for referral to in-person visits suggest that the digital tools provided information and advice that assisted users in making health decisions. These tools may therefore reduce the burden on health care systems during times when resources are limited and may help minimize unnecessary physical interactions that could lead to iatrogenic COVID-19 exposure. However, research on health care use and health outcomes following digital health tool use is needed to conclusively demonstrate this potential.

In addition, individuals who chose to communicate with a remote physician tended to have lower rates of comorbidity. This suggests that these individuals were more likely to have an established relationship with a health care provider whom they consulted regularly and were more likely to require an in-person evaluation and more complex care when presenting with significant symptoms.

This study also highlighted differences in self-reported symptoms between users who reported testing positive for COVID-19 and those who reported testing negative. The symptom most strongly associated with positive testing for COVID-19 was loss of sense of taste or smell (RR 6.66, 95% CI 5.53-7.93). Surprisingly, travel to an area with COVID-19 appeared to be associated with a lower rate of positive testing (RR 0.47, 95% CI 0.23-0.90); however. this estimate is based on a relatively small number of subjects and should therefore be interpreted with caution. As the decision to test or be tested is driven by the presence of risk factors for COVID-19, travel to an area with COVID-19 may appear protective because individuals who were tested due to this risk factor may be less likely to have other stronger predictors of positivity, such as exposure to an individual with COVID-19 or the presence of COVID-19 symptoms. Additional features associated with COVID-19 included other widely recognized COVID-19 symptoms, such as fever and cough. These results emphasize the potential utility of taste and smell as strong signals of COVID-19 positivity in the community setting, and they are similar to results reported in recent studies of self-reported symptoms among individuals who tested positive for COVID-19 [19,20].

Finally, in our study, we found that dry eye (RR 1.87, 95% CI 1.57-2.22) and eye pain (RR 1.61, 95% CI 1.29-2.01) were more common among individuals who reported testing positive for COVID-19. These symptoms were added to the self-assessment to explore the possible link between COVID-19 and eye symptoms as well as the utility of these symptoms in evaluating suspicion of COVID-19. Although some early hospital-based studies reported low rates of ocular symptoms in patients with COVID-19 [20], several other studies have suggested that ocular symptoms are common among individuals with COVID-19 [21-23]. Ocular symptoms have been documented in some cases as the first [24] and even only [25] symptomatic manifestation of COVID-19, and studies have documented positive reverse transcriptase–polymerase chain reaction COVID-19 test results from ocular secretions [23,26]. This study adds to the body of evidence suggesting that the manifestations of COVID-19 include ocular symptoms and that they may be more common symptoms of COVID-19 than generally recognized.

Seen from a wider perspective, these results demonstrate that self-reported symptoms on a digital app can replicate symptoms known to be associated with COVID-19, that they can help distinguish between individuals who test positive and negative for COVID-19, and that they may add to our understanding of symptoms associated with COVID-19. This exemplifies the potential of data generated from digital tools to improve our understanding of the clinical manifestations of COVID-19 and of patient-reported experiences in general.

### Comparison With Prior Work

The potential benefits of digital tools during the COVID-19 epidemic have been noted in multiple health policy commentaries [1-3,19,27]. This stance has been adopted by several leading medical associations, including the American Medical Association and the American Academy of Family Physicians [4,5]. However, there has been limited research to date on the actual usage patterns and impact of these tools during the COVID-19 epidemic. The body of literature on digital health for COVID-19 primarily features perspectives and opinion pieces, guidance papers, and a few studies. These studies tend to be small, focus on a single digital tool, and/or report primarily on survey results of user satisfaction. Two larger studies have provided some additional insight. The first study reported the results of a satisfaction survey of 6194 people following wide-scale deployment of digital tools for COVID-19 education, self-assessment, and symptom monitoring in the Netherlands; high satisfaction rates were reported [11]. The second study reported on the treatment and high satisfaction rates of 4589 patients in China using web-based physician consultation for COVID-19 concerns [13].

The demographic characteristics of digital health users described in this study match previous reports on digital health users. While the use of digital health technologies among seniors has been reported to be increasing [28], users of digital health applications are still predominantly younger adults [29]. The current low level of adoption of digital health by older adults is unfortunate, as digital health technologies have the potential to improve communication and collaboration and promote healthy and independent ageing. Indeed, recent research suggests that digital solutions tailored for older adults improve health management [30]. Barriers to the adoption of digital health by older adults include visual impairment, limitations in dexterity, and lack of self-confidence when using technology. Several solutions are being developed to circumvent these barriers, including the development of voice-based applications and unobtrusive sensors and trackers [31].

### Strengths and Limitations

This study has a number of strengths. First, it uses a large sample of over 71,000 individuals to provide timely information on the use of a number of different digital health tools for managing COVID-19–related concerns. Second, the study reported on the differential use of these tools by these individuals as well as on both self-reported variables and physician-reported disposition and management. Third, the study was able to provide insight into self-reported symptoms of individuals who were tested for COVID-19; the results highlighted the strong link between COVID-19 and loss of sense of smell and taste and added to the body of evidence that ocular symptoms may be a more common feature of COVID-19 than is widely recognized.

The study has a number of limitations as well. The population of this study is not representative of the entire population, and the study uses data from digital tools developed by a single provider. However, the characteristics of the study population and the COVID-19 symptoms reported correspond with those in previous reports on digital health tools [11,20-22]. In addition, data on COVID-19 test status were based on self-reporting and may not accurately capture test results. Furthermore, the data are limited to individuals who used a tool for initial COVID-19 assessment. Tools enabling longitudinal logging and monitoring of symptoms can both provide additional utility to users and improve our understanding of disease progression and the time course of symptoms. Enabling users to provide unsolicited data on their experience may provide important insight as well, as the self-assessment tool described focused on a predefined list of questions. Lastly, while we had data on the disposition of users who chose to consult with a remote physician, the disposition of individuals who did not consult with a remote physician following use of the automated tools is unknown. Research on health care use and health outcomes of digital health users is needed to conclusively demonstrate the utility of these services in assisting individuals with health decision-making and reducing the burden on the health system.

### Conclusion

This study describes the integration of three digital health tools for the direct management of COVID-19–related concerns. The results suggest that automated, data-driven digital solutions as well as remote care provided by a human physician can help provide health information and guidance during an epidemic. In addition, interactions across digital services can provide insight regarding the characteristics of new diseases. The integration of these tools can be an important resource for health care providers and policy makers.

## Appendix

Multimedia Appendix 1 Supplementary information.

## Abbreviations

AI: artificial intelligence
GDPR: General Data Protection Regulation
HIPAA: Health Insurance Portability and Accountability Act
MHS: Maccabi Health Services
RR: relative rate
SARS: severe acute respiratory syndrome
