# Supplementary material for: Characteristics and Symptoms of App Users Seeking COVID-19–Related Digital Health Information and Remote Services: Retrospective Cohort Study
Source: J Med Internet Res. 2020 Oct 20;22(10):e23197. doi: 10.2196/23197 (PMC7609191; doi:10.2196/23197)
Supplement: Multimedia Appendix 1 [file jmir_v22i10e23197_app1.docx]

**COVID-19 self-assessment questionnaire**

The self-assessment was developed by a team of board-certified physicians, and based on guidance issued by the WHO and the CDC. The self-assessment included questions aimed at determining the degree of COVID-19 suspicion, the presence of severe symptoms, and the presence of significant comorbidity. The degree of COVID-19 suspicion was dependent on questions evaluating potential exposure COVID-19, and questions evaluating symptoms characteristic of COVID-19. Severe symptoms were designated as the presence of chest pain, dyspnea, or severe light-headedness. Following the self-assessment, users were provided with one of four recommended actions based on their risk profile: social distancing, quarantine, isolation, and seek immediate medical evaluation. Users were also informed if they were at increased risk for COVID-19 complications; and users with risk factors and symptoms were encouraged to consult a physician.

List of questions:

1. Have you been in contact with anyone who has tested positive for Coronavirus, has symptoms, or is under quarantine for suspected infection? (Y/N)
2. (If No) Have you been in an area with a Coronavirus outbreak in the last 14 days? This can include visiting or living in a place where schools were closed because of the illness.
3. Do you have any of the following heart diseases?
   - High blood pressure
   - Coronary artery disease
   - Congestive heart failure
   - Other heart disease
4. Do you have any of the following chronic lung diseases?
   - COPD
   - Asthma
   - Interstitial lung disease
   - Other lung disease
5. Do you have any of the following?
   - Immunosuppression
   - Diabetes type I
   - Diabetes type II
   - Chronic kidney disease
   - Liver disease
   - Active cancer
   - Past stroke
   - I'm a smoker
   - Morbid obesity
6. Are you feeling so lightheaded that you have trouble standing up or moving around? (Y/N)
7. Are you feeling so short of breath that you have trouble speaking full sentences? (Y/N)
8. Are you experiencing severe chest tightness that limits your ability to take a normal breath? (Y/N)
9. Have you been tested for COVID-19? (Y/N)
   - (If Yes) Did the test show that you have COVID-19? (Y/N)
10. Have you lost your sense of smell or taste? (Y/N)
11. Do you have a stabbing sensation or any pain behind your eyes? (Y/N)
12. Are you having any eye dryness or difficulty focusing your vision? (Y/N)
13. Are you experiencing any muscle pain, unrelated to physical activity? (Y/N)
14. Do you have any joint pain? (Y/N)
15. Are the symptoms you told me about coming and going or changing over time? (Y/N)

**Supplementary Table 1. Frequency of the Top 10 Additional Chief Complaints in Symptom Checker, among COVID-19 positive users (n=292)**

| **Symptom Description** | **%** |
| --- | --- |
| Headache | 6.51 |
| SoreThroat | 5.48 |
| Fatigue | 4.79 |
| Diarrhea | 1.71 |
| Nausea | 1.71 |
| NasalCongestion | 1.03 |
| Runny nose | 1.03 |
| Sweating | 0.68 |
| ThroatIrritation | 0.68 |
| Chills | 0.68 |
